# The efficacy of non‐surgical platelet‐rich fibrin application on clinical periodontal parameters and periostin level in periodontitis: Clinical trial

**DOI:** 10.1111/jcmm.17675

**Published:** 2023-01-23

**Authors:** Sarah Al‐Rihaymee, Maha Sh. Mahmood

**Affiliations:** ^1^ Department of Periodontics College of dentistry, University of Baghdad Baghdad Iraq

**Keywords:** periosteum, periostin, platelet‐rich fibrin, regeneration, root planing

## Abstract

Platelet‐rich fibrin (PRF) has been widely used in regenerative dentistry due to many growth factors produced. Periostin, a matricellular protein, is a reliable marker for tissue regeneration. Periostin is part of the cellular matrix and regulates bone homeostasis. This study aims to explore the efficacy of PRF in improvement of the clinical periodontal parameters as an adjunct to the scaling and root planing and to evaluate periostin level in gingival crevicular fluid (GCF) at baseline, 1‐ and 3‐month recall visits. Fourteen periodontitis patients who met the inclusion criteria were recruited in this study. Two contralateral periodontal pockets with 4–6 mm in depth in each patient were selected. The sites in every participant were randomly allocated into control sites or test sites. In control sites, only conventional scaling and root planing was carried out. In test sites, however, scaling and root planing method and PRF were applied. Periostin level in GCF and clinical periodontal parameters were measured. The test sites revealed greater relative attachment gain (2.614 ± 0.606 mm and 3.321 ± 0.668 mm) than control sites (1.285 ± 0.671 mm and 1.839 ± 0.632 mm) and a significant pocket reduction (2.535 ± 0.664 mm and 3.321 ± 0.668 mm) than the control sites (1.21 ± 0.508 mm and 1.892 ± 0.655 mm) at 1‐ and 3‐month recall visits respectively. In the test sites, level of periostin (48.83 ± 9.3 ng/μl and 98.90 ± 24.94 ng/μl) were greater than periostin levels in the control sites (42.65 ± 7.03 ng/μl and 49.29 ± 15.14 ng/μl) at 1‐ and 3‐month recall visits respectively. In conclusion, the non‐surgical application of PRF as an adjunct to scaling and root planing significantly improved the clinical periodontal parameters through raising periostin level in GCF.

## 1 INTRODUCTION

Destruction of periodontal ligaments, loss of attachment, pocket formation and bone loss are the main characteristics of infectious periodontitis. Thereby, the ultimate goal of periodontal treatment is to eliminate inflammation, prevent periodontal disease progression and promote regeneration of the lost structures.^1^ Regeneration of periodontal tissue is defined as a complete restoration of lost architecture and function of periodontium.^1^, ^2^ So far, blood is the only source that provides essential therapeutic cellular and protein constituents necessary for regeneration, such as red blood cells, white blood cells, plasma and platelets. Specifically, platelets activate and release essential growth factors comprising cytokines, coagulation factors, platelet‐derived growth factor (PDGF), adhesion molecules and transforming growth factor beta (TGF‐β).^3^ In general, growth factors enable the recruitment and activation of fibroblasts, leucocytes and stem cells. Furthermore, growth factors, coagulation factors and cytokines are delivered in the clot by activated platelets. As a result, a complex physiological events resulting in tissue repair and regeneration is established.^4^, ^5^

The outcomes of periodontal therapy have been improved through many surgical approaches which are but not limited to open flap surgery, grafts and guided bone regeneration.^6^ Moreover, the clinical outcomes of periodontal treatment can also be improved using non‐surgical applicants such as plant extract substances,^7^, ^8^ antimicrobial applicants^9^ and laser therapy^10^, ^11^ as adjunct to scaling and root planing. A significant improvement in the wound healing and the regeneration can be achieved by second generation of platelet concentrates which is known as platelet‐rich fibrin (PRF).^12^ PRF contains platelets, undifferentiated cells, growth factors and cytokines which are involved in a matrix like structure. PRF forms a mesh‐like structure allowing the migration and differentiation of cells from nearby region resulting in improvement of wound healing and regeneration of any lost tissue. Furthermore, growth factors in the PRF stimulate osteoid expression and angiogenesis.^13^ PRF has showed positive results in numerous surgical procedures such as sinus lift,^14^ socket fill,^15^, ^16^ periodontal furcation involvement^17^ and intrabony defect.^18^, ^19^, ^20^ The application of PRF in regenerative dentistry has been thoroughly documented by several systematic reviews where soft tissue healing mostly favour over hard tissue healing.^21^, ^22^, ^23^ The data from the current systematic reviews with meta‐analysis reveal that open flap debridement with PRF application has statistically significant clinical improvements in radiographic bone, CAL gain and PPD reduction comparing to open flap debridement alone.^24^ Moreover, the data show that similar results can be achieved when intrabony defects are filled with either bone graft or PRF. In addition, statistically significant enhancements in radiographic bone fill and CAL were noticed by combining PRF and bone graft.^24^ New formed periodontium releases a matricellular protein called periostin which is so called because of its expression mainly in the periodontal ligaments and periosteum of adult mice.^25^ Periostin promotes the migration of fibroblasts and osteoblasts and triggers type I collagen interaction with fibronectin. Therefore, periostin plays a central role in regeneration of the periodontal ligaments and alveolar bone after periodontal surgical procedures.^25^ Moreover, periostin helps in wound healing and remodelling the periodontium.^25^ The non‐surgical use of PRF aids in avoiding the difficulties observed in surgical approaches of the treatment of periodontal defects such as gingival recession, post‐operative pain and discomfort, and prolonged operation time.^26^ In this study, the efficacy of non‐surgical use of PRF adjunctive to the conventional ScRp on clinical periodontal parameters and gingival crevicular fluid (GCF) periostin levels was evaluated.

### 1.1 Hypothesis

*The non‐invasive application of PRF adjunct to scaling and root planing is not significantly effective in improving the clinical periodontal parameters (pocket reduction) when compared with scaling and root planing alone.*

*The non‐invasive application of PRF adjunct to scaling and root planing is significantly effective in improving the clinical periodontal parameters (pocket reduction) when compared with scaling and root planing alone.*

### 1.2 Objectives

1. Measuring the following periodontal clinical parameters: Plaque index (PI), bleeding on probing (BOP), probing pocket depth (PPD) and relative attachment level (RAL) before and after treatment.
2. Measuring GCF periostin levels in periodontitis sites with and without PRF application.

### 1.3 Materials and Methods

**FIGURE 1:**
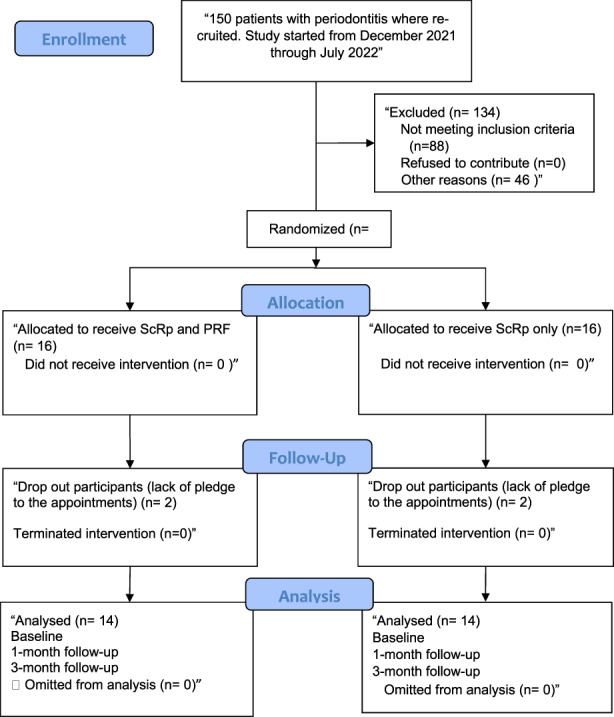
Consort flow chart 2010.

A split mouth randomized control clinical test was the present study design. The study was registered online on ‘www.clinicaltrial.gov’ website with ID of ‘NCT05178771’. A split mouth strategy was employed to assess the response of every patient to two different treatment approaches. As illustrated in consort 2010 flow chart (Figure 1), 14 participants (12 males and two females) were enrolled in the study at department of periodontics at Babylon university. The study was presented from December 2021 to July 2022. Ethical committee, College of Dentistry, University of Baghdad approved this study with protocol number 529,622. All participants were presented with periodontitis according to ‘2017 World Workshop Classification of Periodontal and Peri‐implant Diseases and Conditions’.^27^ The inclusion criteria were two contralateral pockets of 4–6 mm in depth in each periodontitis patient and systemically healthy patients. Whereas, the exclusion criteria were as follows: periodontal therapy for the last 3 months, medical history of systemic diseases such as diabetes, buccal and lingual surfaces of multirooted teeth, patients wearing dental prosthesis, alcoholic patient, teeth with grade II mobility endodontic lesions and untreated caries, and pregnancy or lactation. Coin toss method was used to randomly categorize the contralateral periodontal pockets into control and test sites. Conventional scaling and root planing treatment was used to treat the control sites with no extra adjuncts. Whereas, the test sites were treated with conventional scaling and root planing as well as the application of PRF as an adjunct. The level of periostin in gingival crevicular fluid was measured at baseline, 1‐ and 3‐month recall visits to assess the regeneration of periodontal ligaments and periosteum.

### 1.4 Outcomes

—Primary outcomes: periodontal clinical measurements (pocket depth) set as a primary outcome of the study.

—Secondary outcomes: Periostin levels in GCF set as secondary outcome of the study.

Clinical periodontal parameters, including PPD, RAL, PI and BOP^28^ were taken at baseline and 1‐ and 3‐month recall visits. A periodontal probe (UNC‐15) was used to take the periodontal clinical measurements from six sites of the teeth which were distolingual, mid‐lingual, mesiolingual distobuccal, midbuccal and mesiobuccal.

For standardization and alignment exercise and to get an acceptable level of agreement (>0.75) of all clinical periodontal parameters, inter‐ and intra‐examiner calibrations were used to assess the accuracy and reproducibility of the examiner for clinical periodontal parameters (PPD and RAL). Inter‐examiner calibration scores were recorded by the researcher with the help of a skilled examiner for eight subjects.^29^ To calibrate intra‐examiner, the researcher measured the clinical periodontal parameters for five patients twice with 2 h interval between the two measurements.

### 1.5 Sample size calculation

Mean of pocket reduction was used to calculate the sample size. Özcan et al. calculated the means of pocket reduction of test and control groups which were 2.57 ± 0.75 and 0.93 ± 0.49 mm respectively.^26^ For sample size calculation, G power software (v. 3.1.9.6) was used with 95% confidence interval and margin of error of 5%. The estimated size effect was 2.58. The total sample size for periodontitis sites was 24, which was rounded to 28 sites to compensate for dropout of samples. Hence, 14 patients (two sites in each patient) were recruited in this study. Accordingly, the sites were calculated to be 14 control sites and 14 test sites. The allocation ratio of 1:1 was followed.

### 1.6 GCF sampling

GCF collection was performed to measure the level of periostin at baseline, 1 month and 3 months. GCF was collected from control and test sites using PerioCol papers (PerioCol, Orafollow, USA) by intracrevicular method and the volume of GCF samples was measured using Periotron model 3,046,000. The preferable time for GCF collection is 9 AM–11 AM to avoid any diurnal variance.^26^ To avoid blood contamination of PerioCol paper during GCF collection, fluid collection was carried out next day after measuring the clinical periodontal parameters. Moreover, to prevent saliva contamination, sites were dried by blast of air and isolated with cotton rolls. Then, the PerioCol paper was inserted in the examined site in the gingival crevice until slight resistance was sensed. After 30 s, PerioCol paper was gently removed and immediately placed between the jaws of periotron. After taking the score, the PerioCol paper was transferred to the Eppendorf microtube with phosphate buffered saline and stored in ‐42°C low temperature freezing. The standardization was preserved before every sample collection by the calibrated Periotron device. To increase the accuracy of sampling, two PerioCol papers with GCF sample were collected from each site. Every single PerioCol paper was placed in a separate microtube and stored until all samples were collected and ready to be analysed in the laboratory.

### 1.7 Clinical intervention

**FIGURE 2:**
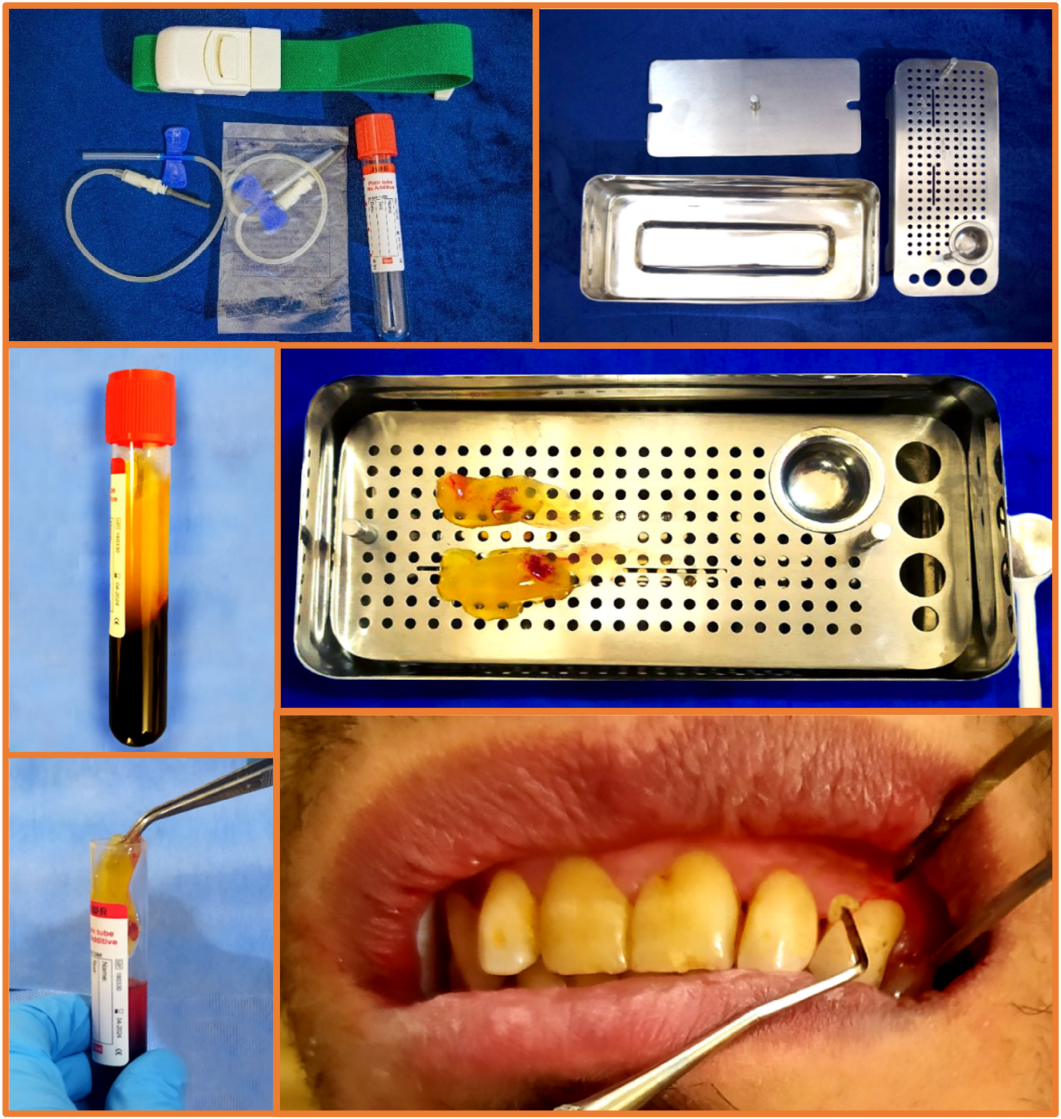
Equipments of for PRF preparation, its preparation and application in periodontal pocket.

A split mouth technique in which two quadrants in every participant with periodontal pocket of 4–6 mm in depth were chosen, was performed. All participants received a written informed consent which was acknowledged and signed. In the initial phase, all patients received instructions on the use of oral hygiene aids and the correct way for brushing their teeth. The first visit also was included recording of indices (PI, BOP, PPD and RAL) after taking impression for costume acrylic stent construction for RAL measurement. GCF samples were collected at baseline and 1‐ and 3‐month follow‐up visits. The first visit also involved scaling procedure for the entire dentition using ultrasonic device. For oral hygiene assessment and other periodontal procedures, the patients were recalled after 7 days. For every participant, periodontal pockets in two contralateral quadrants were selected. Coin toss method was used to randomly categorize the sites into control and test sites. Scaling and root planing technique was used to treat the control sites only, whereas the scaling and root planing in addition to PRF were employed to treat the test sites. Scaling and root planing technique was carried out to eliminate the subgingival and calculus from the base of the pocket until the root surface was felt smooth, clean and hard using standard curettes. After the scaling and root planing, saline solution was used to irrigate the pockets. After PRF preparation explained in the next section, a fibrin clot formed in the centre of the tube between the red mass (RBCs) at the base of the tube and plasma at the top. Then, the fibrin clot was detached from the red blood corpuscles using tweezer and surgical scissor and transferred immediately on a sterile PRF box. Gently compressing the PRF for 10 min to thin out the PRF to 1 mm thick. The PRF pieces were placed into the base of the periodontal pocket up to the marginal gingiva to fill the pocket (Figure 2). The clinical periodontal parameters were measured in 1‐ and 3‐month recall visits by an experienced dentist to achieve the blindness of this study.

### 1.8 PRF preparation protocol

The common protocol of PRF preparation was followed.^18^ In order to collect blood from the patient, antecubital vein venipuncture was made by butterfly needle connected to 10 ml sterile vacuum tube (free from any anticoagulants). Immediately after collecting intravenous blood, the tube was centrifuged at 3000 revolutions per minute for 10 min. To assure reproducibility of data and minimization of confusion in the field of PRF, it has been advised to report relative centrifugation force (RCF).^30^ In fact, three main locations RCF can be measured. RCF‐min, RCF‐max and RCF‐clot are calculated at the upper, bottom portion of PRF tubes and location at which the PRF clot is formed respectively.^31^ RCF‐clot is liable to centrifugation time. However, because RCF‐max is unalterable by the centrifugation time,^31^ the international standard method promotes utilizing RCF‐max. Therefore, RCF‐clot produce less accurate outcomes, and is not commonly reported internationally.

The needed parameters key for the report of RCF^30^ are (Table 1):

1. Centrifugation model.
2. Rotor angulation.
3. Radius at the bottom of the tube.
4. Revolutions per minute (rpm) and time.
5. RCF value calculated at RCF‐max; and
6. Size and composition of tubes used to produce PRF.

**TABLE 1:** Required parameters for the report of RCF.

Device	IntraSpin
Rotor angulation	33
Distance to rotor in mm	80 mm
Revolutions per minute	3000 rpm
Time (min)	10
RCF‐max	805 g
Size of tubes	10 ml
Composition of tubes	Plain glass tube without chemical additive

RCF (× g) can be calculated by the following formula:

$$\mathrm{RCF}\;\max=11.18\times r\times {\left(N/1,000\right)}^{2}$$

where r denotes the radius in millimetres measured from the centre of the rotor to the bottom of the tube, and N is the rotor speed in rpm.^32^ In this study, the parameters used to calculate the RCF‐max were as follows:

### 1.9 Post‐operative instructions

The patients were asked not to brush their teeth during the first day after the procedure to avoid PRF dislodgment. But patients were encouraged to continue brushing their teeth after the next day. No mouth wash or prescription was advised following the therapy. The recall visits were scheduled at 1 and 3 months to measure periostin level in the gingival crevicular fluid. At every recall visit, oral hygiene instructions and motivation were presented fitting to personal needs.

### 1.10 Biochemical analysis

The measurement of periostin GCF levels was taken using enzyme‐linked immunosorbent assay (ELISA) method. ELISA used in the laboratory was Human periostin ELISA kit. Manufacturer's instructions were followed to perform the measurements. Periostin the intra‐assay coefficient of variation (CVS) was <2.8%–4.6%, inter‐assay CVS were < 10% and the analytic sensitivity of these assays yielded 0.251 ng/ml.

Antibody specific for human periostin coated on a 96‐well plate and 48‐well plate were used. Samples and standards of periostin were pipetted into the wells. The immobilized antibody bounded periostin in samples to the wells were washed. Biotinylated antibody and streptavidin were pipetted into the wells were washed again. A tetramethylbenzidine one‐step substrate solution was added to the wells. The colour developed depending on the levels of periostin. Finally, stop solution was added, and the formation of yellow colour was measured at 450 nm using a spectrophotometer.

### 1.11 Statistical analysis

The Shapiro test was used to determine the normal distribution of data. A descriptive analysis to calculate mean and standard deviation (Mean ± SD) was performed for all clinical periodontal and biochemical parameter. The difference in clinical periodontal parameters between test and control groups was determined using Mann–Whitney *U* test. A comparison of recall visits in intragroup was made using Wilcoxon test. Independent *t* test was performed to calculate the significant differences between test and control groups regrading periostin levels. Repeated measure anova test was performed to determine the differences between recall visits in intragroup. In the overall analysis, *p* ˂ 0.05 was considered as significant.

## 2 RESULTS

Fourteen participants comprised 12 males and two females including 24 sites were studied. The illustration of PRF and ScRp that were locally applied are shown in the Figure 2. The mean clinical periodontal parameters measured at baseline, 1 and 3 months are shown in Table 2. The results shown in Table 2 indicated no noticeable difference among baseline clinical periodontal parameters between groups. Significant changes in clinical periodontal parameters (PLI, BOP) were produced as both treatment forms showed. In the ScRp+PRF group, the results showed significant low level of PPD and RAL compared to those in the ScRp group at the 1‐ and 3‐month recall visits (*p* < 0.01).

**TABLE 2:** Clinical periodontal parameters of control and test groups at baseline and 1‐ and 3‐month recall visits.

Clinical outcomes	Time	Test		Control		Control and test groups
		Mean ± SD	*p*	Mean ± SD	*p*	*p*
PI	Baseline^a^	1.71 ± 0.825	0.001^a,b^	0.93 ± 0.730	0.001^a,b^	0.454
	1 month^b^	0.36 ± 0.497	0.083^b,c^	0.36 ± 0.497	0.083^b,c^	1.000
	3 month^c^	0.14 ± 0.363	0.001^a,c^	0.14 ± 0.363	0.001^a,c^	1.000
BOP	Baseline^a^	0.357 ± 0.497	0.025^a,b^	0.357 ± 0.497	0.025^a,b^	1.00
	1 month^b^	0.00 ± 0.00	1.00^b,c^	0.00 ± 0.00	1.00^b,c^	1.00
	3 month^c^	0.00 ± 0.00	0.025^a,c^	0.00 ± 0.00	0.025^a,c^	1.00
PPD	Baseline^a^	5.429 ± 0.646	0.001^a,b^	4.929 ± 0.730	0.001^a,b^	0.94
	1 month^b^	2.893 ± 0.349	0.001^b,c^	3.714 ± 0.611	0.001^b,c^	**0.01**
	3 month^c^	2.107 ± 0.289	0.001^a,c^	3.036 ± 0.535	0.001^a,c^	**0.000**
RAL	Baseline^a^	7.929 ± 0.730	0.001^a,b^	7.357 ± 1.081	0.001^a,b^	0.178
	1 month^b^	5.314 ± 0.649	0.001^b,c^	6.071 ± 1.035	0.004^b,c^	**0.044**
	3 month^c^	4.607 ± 0.684	0.001^a,c^	5.518 ± 0.868	0.001^a,c^	**0.006**

*Note*: The bolded numbers refer to statistically significant differences (*p* < 0.05 and *p* < 0.01).

^a,b^Comparision of the baseline and 1 month,^, b,c^1 and 3 months after treatment and ^a,c^Baseline and 3 months.

**FIGURE 3:**
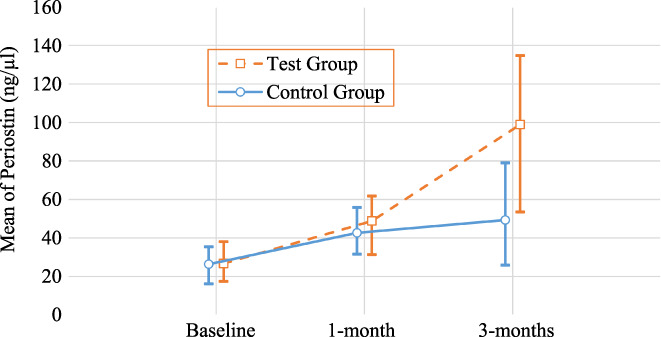
The mean values of periostin levels in GCF (ng/μl) at baseline, 1 month and 3 months recall visits in test and control sites (Vertical bars denote 0.95 confidence intervals).

**FIGURE 4:**
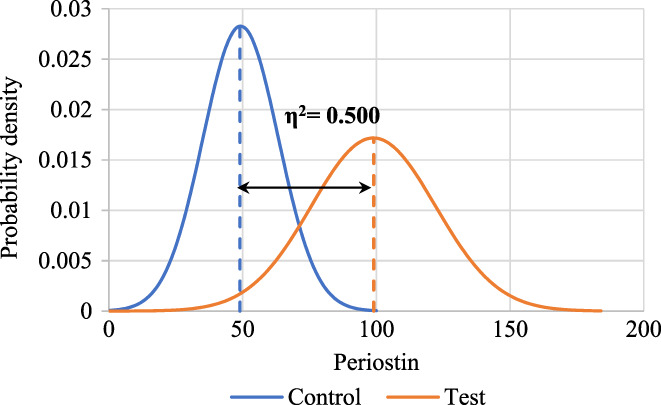
Effect size determination for periostin level among study groups using partial ETA square (*η*2).

PPD reduction, RAL gain and their comparisons at the 1‐ and 3‐month recall visits are shown in Table 3. The ScRp+PRF group at the 1‐ and 3‐month recall visits shows very high PPD reduction and RAL gain compared to those in the ScRp group (*p* < 0.01). The biochemical parameter and its comparisons are presented in Table 4. The results shown in Table 4 and Figure 3 showed no substantial differences in the periostin levels between ScRp+PRF and ScRp groups at baseline. The results also showed that periostin levels (48.83 ± 9.3 ng/μl, 98.90 ± 24.94 ng/μl) in the ScRp+PRF group were significantly higher than periostin levels (42.65 ± 7.03 ng/μl, 49.29 ± 15.14 ng/μl) in the ScRp group at 1‐ and 3‐month recall visits respectively. The changes of the biochemical parameter in control group and test group are shown in Figure 3 at baseline and 1‐ and 3‐month recall visits. A large size effect (*η* ^2^ = 0.500) between test and control groups among periostin level was noticed as shown in Figure 4.

**TABLE 3:** The change in the clinical periodontal parameters (PPD reduction and RAL gain) of control and test groups at 1‐ and 3‐month recall visits.

Clinical periodontal parameters	Time (months)	Test	Control	*p*‐Value
Mean pocket reduction (mm)	1	2.535 ± 0.664	1.21 ± 0.508	**0.001**
	3	3.321 ± 0.668	1.892 ± 0.655	**0.000**
Mean RAL gain (mm)	1	2.614 ± 0.606	1.285 ± 0.671	**0.000**
	3	3.321 ± 0.668	1.839 ± 0.632	**0.000**

**TABLE 4:** The biochemical parameter (periostin) Mean ± SD of control and test groups at baseline and 1‐ and 3‐month recall visits.

Biochemical parameter	Time	Test		Control		Test and control groups
		Mean ± SD	*p*	Mean ± SD	*p*	*p*
Periostin (ng/μl)	Baseline^a^	26.35 ± 6.53	**0.000** ^**a,b**^	26.70 ± 6.51	**0.000** ^**a,b**^	0.08
	1 month^b^	48.83 ± 9.3	**0.000** ^**b,c**^	42.65 ± 7.03	0.344^b,c^	0.058
	3 months^c^	98.90 ± 24.94	**0.000** ^**a,c**^	49.29 ± 15.14	**0.000** ^**a,c**^	**0.00**

*Note*: The bolded numbers refer to statistically significant differences (*p* < 0.05 and *p* < 0.01).

^a,b^Comparision of the baseline and 1 month,^, b,c^1 and 3 months after treatment and ^a,c^Baseline and 3 months.

## 3 DISCUSSION

PRF provides ample amounts of platelets, cytokines and growth factors which are considered the main prerequisite for regeneration of the lost periodontal tissue resulting from periodontitis.^21^ PRF stimulates migration, proliferation and differentiation of cells causing bone and periodontal ligaments regeneration. Periostin is mostly expressed in periodontium and considered as a main regulator of periodontal tissue homeostasis and regeneration.^33^, ^34^ Fibroblast cells release periostin whose level decreases in diseased periodontium.^35^ Increased periostin level after periodontal therapy results in stimulation of cells migration, proliferation and differentiation.^35^ Thus, periostin plays an essential role in periodontal tissue regeneration following periodontal treatment. Periostin level in GCF was measured at baseline and 1‐ and 3‐month recall visits to assess the regeneration effect of PRF of periodontium.

GCF with its mechanical flow effects of cleansing the pocket is regarded as an excellent media for evaluating the released growth factors from platelet concentrate constituents at different time periods. GCF was collected at baseline, and 1‐ and 3‐month recall visits to measure the level of periostin throughout the regeneration process. In the control and test sites, BOP and plaque control scores were optimal (Table 2).

The results shown in Table 3 indicated that PPD reduction (2.535 ± 0.664 mm, 3.321 ± 0.668 mm) and RAL gain (2.614 ± 0.606 mm, 3.321 ± 0.668 mm) in the ScRp + PRF group were greater than PPD reduction (1.21 ± 0.508 mm, 1.892 ± 0.655 mm) and RAL gain (1.285 ± 0.671 mm, 1.839 ± 0.632 mm) in the ScRp group, at the 1 and 3 months respectively.

The findings of this study were compared with studies that utilized PRF with non‐surgical approach and studies that used PRF with surgical methods. The results of this study were very similar compared to the results of studies that used PRF with non‐surgical as well as the studies that used PRF with surgical. Özcan, E.,et al showed that the average of PPD at sites treated with ScRp+PRF were 2.57 ± 0.75 mm at 3 months.^26^ Lohi et al. reported that the average reduction in PD of patients for whom PRF with bioactive grafts was applied was 3.38 ± 1.06 mm.^17^ Patel et al. showed that the reduction of PD after 6 months were 3.0 ± 1.70 mm.^20^ Pradeep et al. stated that the average reduction of PPD was 3.03 ± 1.16 mm.^18^ These similarities emphasize the extreme and incomparable power of the PRF biological effects in both the surgical and non‐surgical techniques.

The results indicated that the lowest concentration of periostin in test sites (26.35 ± 6.53 ng/μl) and control sites (26.70 ± 6.51 ng/μl) was at baseline (Table 4). The reason behind being the lowest level of periostin specifically at baseline reflects the destructive effect of inflammation on periodontium.^35^ Periostin concentration gradually increased after periodontal therapy.^35^ It was observed that the level of periostin in GCF in test sites (48.83 ± 9.3 ng/μl, 98.90 ± 24.94 ng/μl) was more than those in control sites (42.65 ± 7.03 ng/μl, 49.29 ± 15.14 ng/μl) at 1 and 3 months after application of PRF. New formed periodontal ligaments and periosteum release intense amounts of periostin throughout their formation. Therefore, periostin level increases after periodontal therapy. Park et al. stated that periostin could be used as a biomarker of periodontal regeneration in a tissue engineering study in rats.^36^

For years, the importance of PRF tubes has been ignored while the choice of PRF centrifugation devices has gotten the primary consideration of clinicians. However, the recent research has showed that PRF tubes obviously have a larger impact on the PRF clots than centrifugation devices.^37^ A various commercial glass tubes (with or without chemical additives) and silica‐coated plastic tubes have showed different degrees of success. Specifically, consuming tubes containing chemical additives for the production of PRF have potential risks which can alter tissue regeneration at implantation site. For example, silica‐coated plastic tubes generate a recognizable kind of fibrin matrix regarding platelet distribution and contamination by silica particles. Since silica coatings have a great affinity on the walls of cell membrane, it is able to trigger apoptosis and reduce cell proliferation.^38^ Likewise, silicone added to glass tubes has a major impact on increasing inflammatory reactions in humans along with delays in the formation of clot following standard centrifugation protocols.^37^ Glass tubes, on the other hand, produce larger PRF membranes than plastic tubes and silica‐coated plastic tubes.^38^ Size of clot diverse as much as 200% when different tube types were used, whereas it was negligeable when different centrifugation devices were utilized. Furthermore, chemical‐free glass tubes are more favourable than the whole 10‐day period of study.^39^

This study is associated with some practical limitations. for instance, PRF is a slippery material so it can be easily detached out from the pocket throughout its placement. Moreover, narrow periodontal pockets were challenging. To overcome this obstacle, repeated small size pieces of PRF were applied. To prevent mechanical dislodgment of PRF from the pocket, the patients were informed to stay away from brushing their teeth during the first day following the therapy.

## 4 CONCLUSIONS

The non‐surgical application of platelet‐rich fibrin (PRF) as adjunct to the conventional scaling and root planing can significantly improve the healing process through boosting periostin level. Moreover, reduction of the treatment time and decrease in the patient discomfort and post‐operative pain, are considered the major advantages of presented technique over surgical techniques.

## AUTHOR CONTRIBUTIONS

**Sarah Alrihaymee:** Data curation (lead); formal analysis (lead); funding acquisition (lead); investigation (lead); methodology (lead); writing – original draft (lead); writing – review and editing (lead). **Maha Sh Mahmood:** Project administration (equal); supervision (lead).

## CONFLICT OF INTEREST

All the authors certify that they have no conflict of interest to disclose in relation to the subject matter or materials discussed in this study.

## Data Availability

The data that support the findings of this study are available on request from the corresponding author. The data are not publicly available due to privacy or ethical restrictions.
